# Muscular Exertion as a Cause of Albuminuria

**Published:** 1876-06

**Authors:** 


Muscular Exertion as a Cause of Albuminuria.—Mr. Benj. Duke, writing to the London *Lancet*, says: "When a student at Guy's, I entered for the mile race in the United Hospital Sports. Two days previous to the race I ran a mile. The same evening, happening to be reading a work on the urine, describing how to test the same, I thought, by way of illustration, to test my own. My horror may be imagined on finding it to be nearly solid with albumen. I did not feel ill at the time, and it disappeared within twenty-four hours. The next day but one previous to the race I tested my urine, and found it healthy, and again ran a mile, and on examining some urine passed immediately after, found it nearly solid with albumen, which again totally disappeared in about twenty-four hours, and may therefore be fairly attributed to exercise; for it is now nearly nine years since I have found any."

